# Supplementary material for: Multi-day rhythms modulate seizure risk in epilepsy
Source: Nat Commun. 2018 Jan 8;9:88. doi: 10.1038/s41467-017-02577-y (PMC5758806; doi:10.1038/s41467-017-02577-y)
Supplement: Supplementary file 1 — Supplementary Information [file 41467_2017_2577_MOESM1_ESM.pdf]

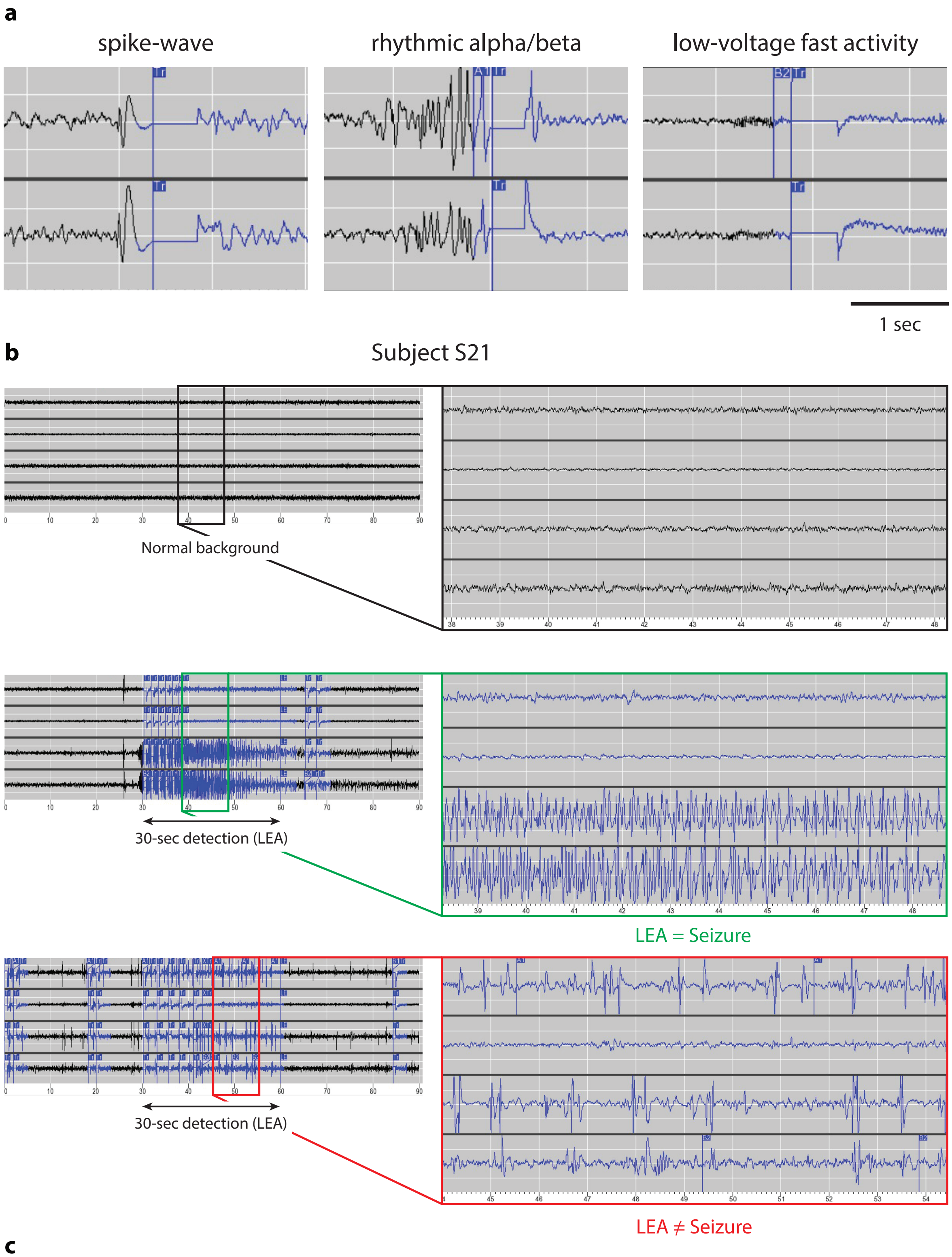

c

$$\mathrm{PPV} = 100 \times \frac{(\#\ \mathrm{LEA} = \mathrm{Seizure})}{(\#\ \mathrm{LEA} = \mathrm{Seizure}) + (\#\ \mathrm{LEA} \neq \mathrm{Seizure})}$$

Supplementary Figure 1. Brain activity detected by the RNS System. (**a**) Examples of epileptiform patterns that the RNS System can detect for clinical purposes, including spike-wave discharges, rhythmic alpha/beta frequencies, and low-voltage fast activity. Hourly counts of detections of these patterns, not the waveforms themselves, constitute the raw data analyzed in this study. Vertical lines labeled ‘Tr’ denote stimulation delivered in response to abnormal activity detected with subject-specific algorithms (labeled ‘A1’ and ‘B2’ in two right panels, label not shown in left panel) based on line length and/or bandpass tools. (**b**) Representative EEGs from subject S21. Numbers on x-axis are time in seconds. Top panels show normal background activity. Middle panels show abnormal activity meeting detection criteria in a sustained manner (here, >30 seconds) to constitute Long Epileptiform Activity (LEA); by inspection, this LEA represents an electrographic seizure. Bottom panels show EEG recorded on a different day in which a prolonged detection event (LEA) occurs due to abundant interictal spiking that does not constitute an electrographic seizure. (**c**) Formula for calculating Positive Predictive Value (*PPV*) for LEA representing an electrographic seizure. Seizure timing analysis in this study was performed only for epochs with *PPV* greater than 90% and averaging 98%.

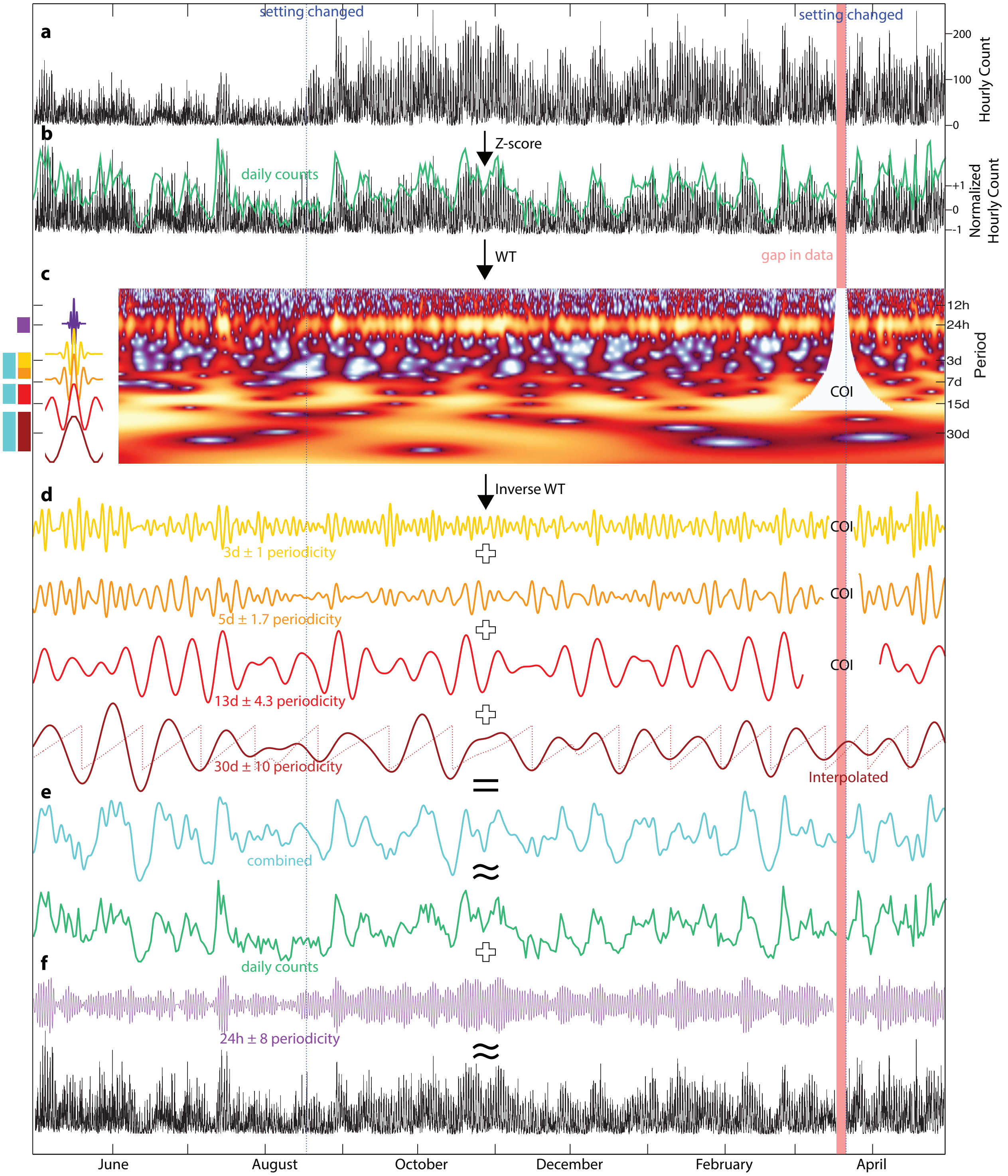

Supplementary Figure 2. Illustration of signal processing methods using one year of data in one subject. (**a**) Raw hourly counts shown as a continuous time-series with two detection settings changes (note that the absolute number of counts shifts up after the first change, reflecting more sensitive detection settings) and one recording gap in data (pink box). (**b**) Hourly counts are normalized by z-scoring individual segments between detection settings changes. This results in attenuation of the shifts in absolute counts observed in (**a**) and decreases edge

artifacts after Wavelet Transform (WT). Green contour corresponds to hourly counts averaged over one calendar date. (**c**) Power spectrogram after Morlet WT. Wavelets at different scales (i.e. periods) are represented on the left side of **c** with corresponding color-code. Power is concentrated at multiple peak periodicities (12h, 24h, 13d and 30d). Periodograms presented in the study are an average of the power spectrogram over time (x-axis). Missing data was interpolated only for periods five times longer than the gap, resulting in continuous power time-series for long periods (here above 20 d) and a gap for shorter periods that depended on the corresponding Cone Of Influence (COI, one full cycle of data at this period). (**d**) Band-pass filtered data obtained by the inverse WT of wavelet coefficients at four selected periods (± 33.3%) are shown here to illustrate how the original data can be reconstructed by combining oscillations at different periods. The dashed dark-red curve oscillates between $-\pi$ and $+\pi$ and represents the instantaneous phase derived by WT and used for phase analysis in this study. (**e**) The cyan curve is the combination of all above curves and is a good approximation of the daily counts. (**f**) Adding the curve with a 24-hour period approximates the original data.

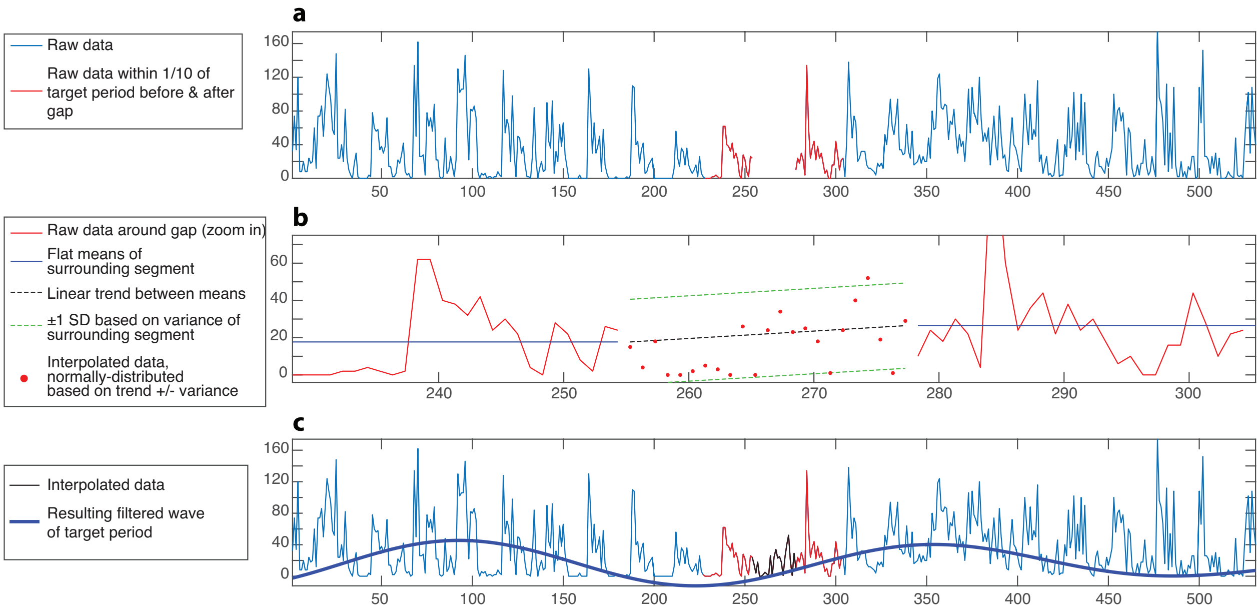

Supplementary Figure 3. Interpolation method for short gaps in data (relative to the period of interest). **(a)** Sample of continuous raw data with a short gap in recording in the middle. (**b**) Section zoomed in around the gap (data in red in top panel), and the kriging methodology (see legend). **(c)** Same data as in **a**, with the interpolated data in black, and the reconstructed oscillation at the period of interest in blue. Note that interpolated data is always less than 20% of the cycle duration of the frequency of interest, to ensure conservative estimates with minimal potential for artifact in subsequent power analyses.

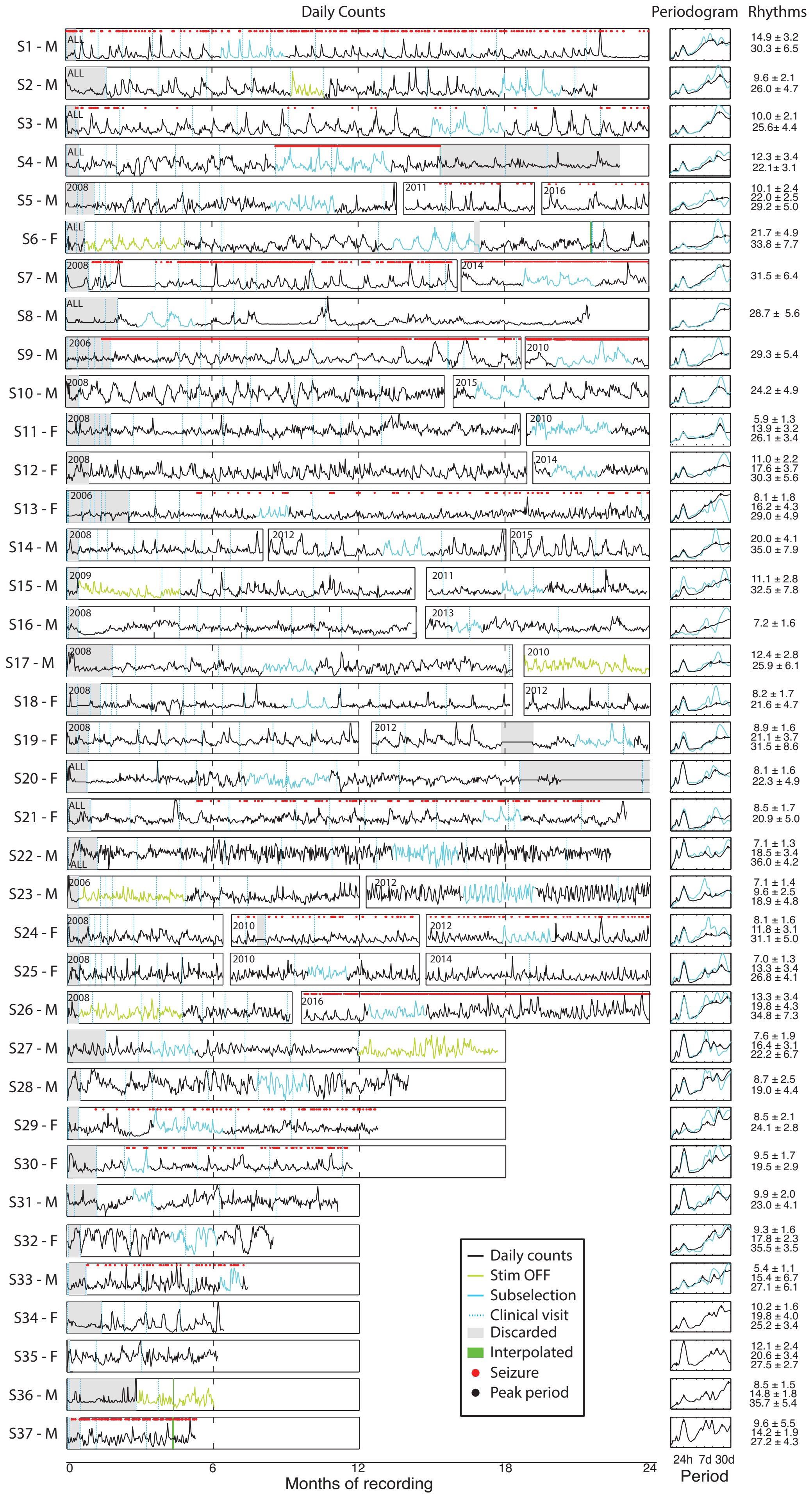
Daily Counts
Periodogram
Rhythms
S1 - M
S2 - M
S3 - M
S4 - M
S5 - M
S6 - F
S7 - M
S8 - M
S9 - M
S10 - M
S11 - F
S12 - F
S13 - F
S14 - M
S15 - M
S16 - M
S17 - M
S18 - F
S19 - F
S20 - F
S21 - F
S22 - M
S23 - M
S24 - F
S25 - F
S26 - M
S27 - M
S28 - M
S29 - F
S30 - F
S31 - M
S32 - F
S33 - M
S34 - F
S35 - F
S36 - M
S37 - M
14.9 ± 3.2
30.3 ± 6.5
9.6 ± 2.1
26.0 ± 4.7
10.0 ± 2.1
25.6± 4.4
12.3 ± 3.4
22.1± 3.1
10.1 ± 2.4
22.0 ± 2.5
29.2 ± 5.0
21.7 ± 4.9
33.8 ± 7.7
31.5 ± 6.4
28.7 ± 5.6
29.3 ± 5.4
24.2 ± 4.9
5.9 ± 1.3
13.9 ± 3.2
26.1 ± 3.4
11.0 ± 2.2
17.6 ± 3.7
30.3 ± 5.6
8.1 ± 1.8
16.2 ± 4.3
29.0 ± 4.9
20.0 ± 4.1
35.0 ± 7.9
11.1 ± 2.8
32.5 ± 7.8
7.2 ± 1.6
12.4 ± 2.8
25.9 ± 6.1
8.2 ± 1.7
21.6 ± 4.7
8.9 ± 1.6
21.1 ± 3.7
31.5 ± 8.6
8.1 ± 1.6
22.3 ± 4.9
8.5 ± 1.7
20.9 ± 5.0
7.1 ± 1.3
18.5 ± 3.4
36.0 ± 4.2
7.1 ± 1.4
9.6 ± 2.5
18.9 ± 4.8
8.1 ± 1.6
11.8 ± 3.1
31.1 ± 5.0
7.0 ± 1.3
13.3 ± 3.4
26.8 ± 4.1
13.3 ± 3.4
19.8 ± 4.3
34.8 ± 7.3
7.6 ± 1.9
16.4 ± 3.1
22.2 ± 6.7
8.7 ± 2.5
19.0 ± 4.4
8.5 ± 2.1
24.1 ± 2.8
9.5 ± 1.7
19.5 ± 2.9
9.9 ± 2.0
23.0 ± 4.1
9.3 ± 1.6
17.8 ± 2.3
35.5 ± 3.5
5.4 ± 1.1
15.4 ± 6.7
27.1 ± 6.1
10.2 ± 1.6
19.8 ± 4.0
25.2 ± 3.4
12.1 ± 2.4
20.6 ± 3.4
27.5 ± 2.7
8.5 ± 1.5
14.8 ± 1.8
35.7 ± 5.4
9.6 ± 5.5
14.2 ± 1.9
27.2 ± 4.3
Daily counts
Stim OFF
Subselection
Clinical visit
Discarded
Interpolated
Seizure
Peak period
0
6
12
18
24
Months of recording
24h 7d 30d
Period

Supplementary Figure 4. Individual (N=37) daily counts and periodograms. Subject number and gender (M: male, F: female) shown on left. S29 has catamenial seizures. Normalized daily counts shown for the total recording length (subjects with < 2 years of data), or at selected times including the first few months (subjects with 2-10 years of data). Responsive stimulation was OFF for a period of time (green) in a subset of subjects, who continue to show multidien rhythms during these times. Periodograms on the right show narrow ultradian and circadian peaks in all subjects. Note that multidien peaks are readily visible for selected periods of time (blue periodograms), but tend to flatten when calculated on entire time-series (black, up to 10 years, not all raw data visible beyond 2 years), due to some degree of variability in periodicity. Discarded data (grey boxes) included the first few months after RNS System implantation (until stable detection settings were achieved) for all subjects, as well as gaps in recordings for some subjects. Interpolated data was minimal and done only if a gap was < 20% of a given period (e.g. narrow green band in last months for S6). Seizures (red dots) were reliably detected in 14 subjects (S1, S3-5, S7, S9, S13, S21, S24, S26, S29-30, S33 and S37), and, for subjects with moderate seizure rate, alignment of seizures near peaks of IEA is apparent (e.g. S13 and S24, but also others). Average period length ± SD of peak periodicities (black dots in periodograms) indicated on the right.

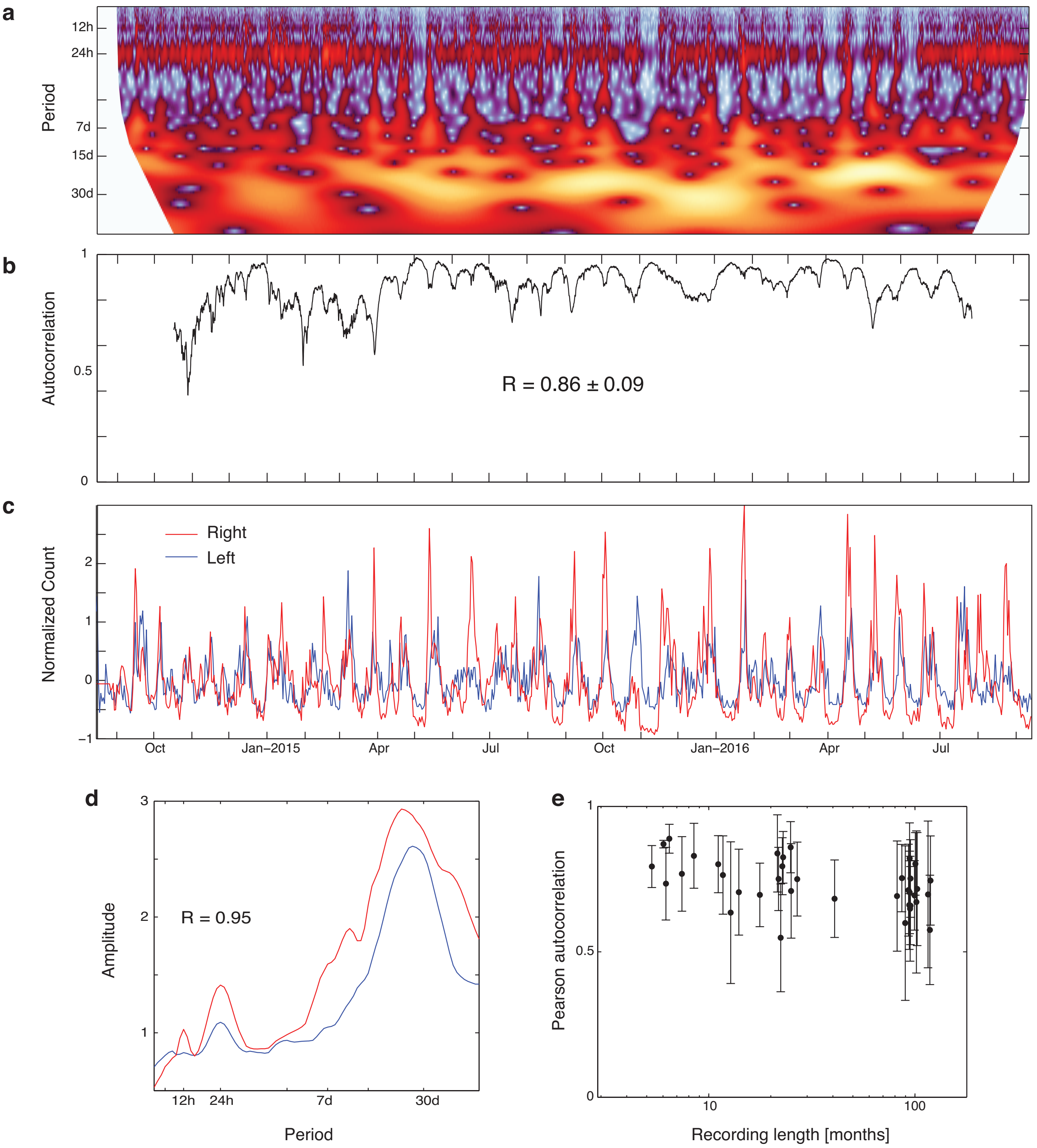

Supplementary Figure 5. Stability of multidien rhythms over time and in anatomically distinct seizure foci. (**a**) Representative spectrogram for one subject (same subject as in Fig. 1) over 24 months. (**b**) Autocorrelation Pearson coefficients between average periodogram and spectrogram at each time-point where power was calculated for all frequency bins, excluding cone of influence. (**c**) Normalized daily counts derived from left and right hippocampal electrodes in the same subject. Of note, absolute counts recorded from these electrodes differed by one order of magnitude, but after normalization, similar rhythms are readily observable. (**d**) Periodograms for corresponding daily counts in (**c**) revealing high correlation between sites on opposite hemispheres. (**e**) Autocorrelation for all subjects (N=37) versus length of recording reveals relative stability over time, even for longer recordings (x-axis logarithmic).

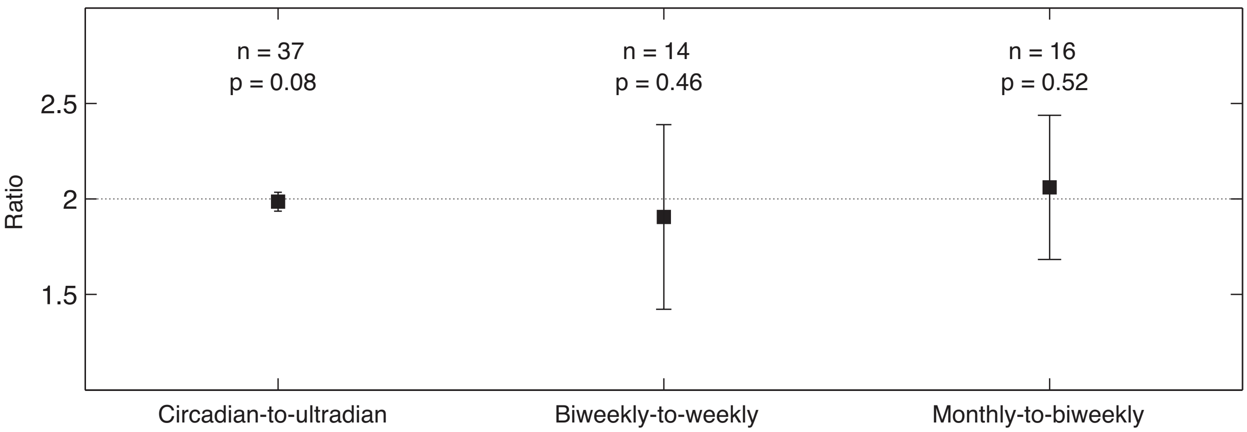

Supplementary Figure 6. Peak periodicities and harmonics. Ratios of peak periods calculated at the individual level for a subject with multiple peaks within six predefined bands (4–17 h, 18–30 h, 4–9 d, 10–16 d, and 25–35 d). For circadian-to-ultradian and multidien rhythms, the ratios are not different from the expected average of 2 (Wilcoxon test, $p > 0.05$), suggesting a harmonic structure to the periodicity (N in Figure).

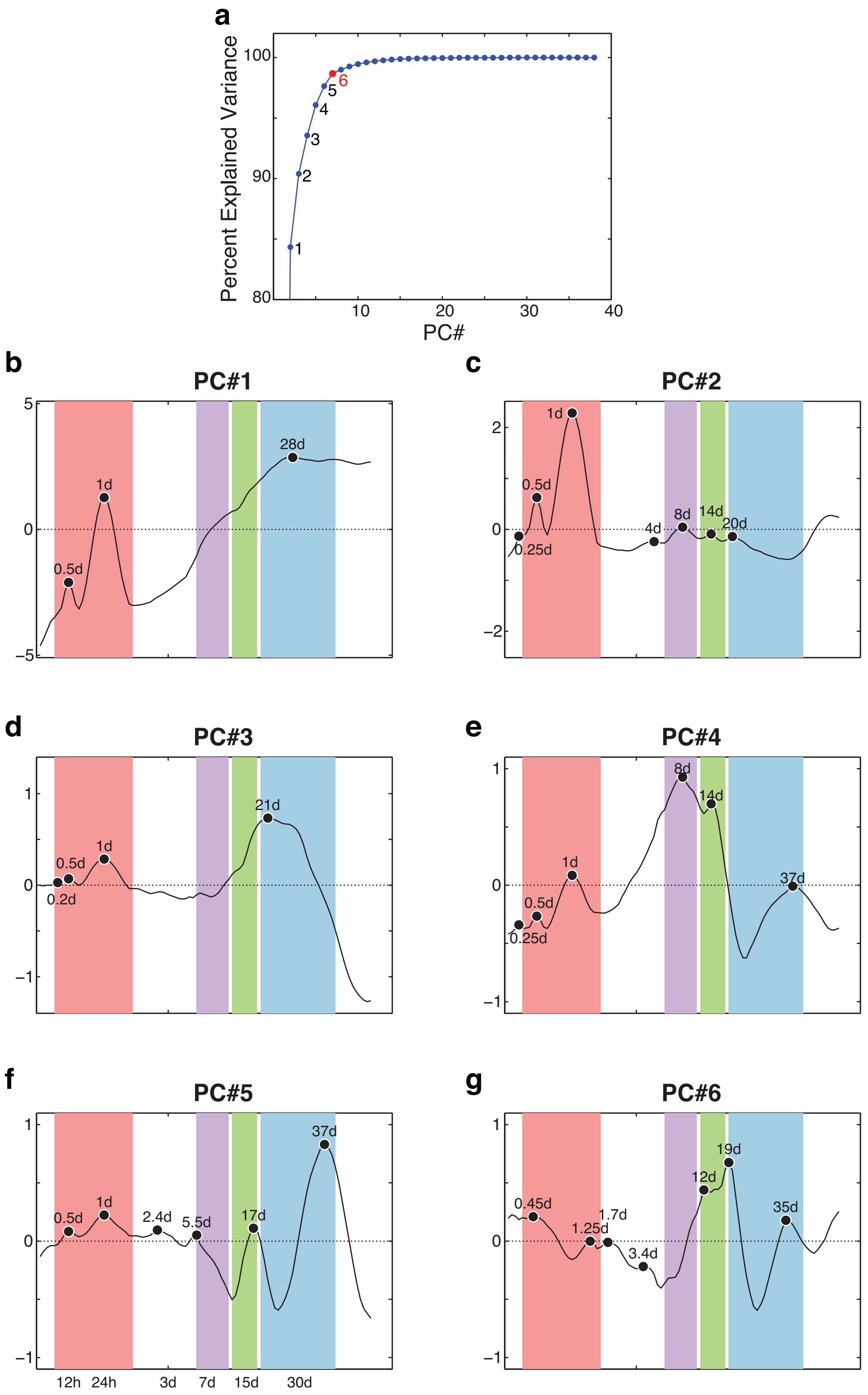

a
Percent Explained Variance
PC#
b
PC#1
0.5d
1d
28d
c
PC#2
0.25d
0.5d
1d
4d
8d
14d
20d
d
PC#3
0.2d
0.5d
1d
21d
e
PC#4
0.25d
0.5d
1d
8d
14d
37d
f
PC#5
0.5d
1d
2.4d
5.5d
17d
37d
12h
24h
3d
7d
15d
30d
g
PC#6
0.45d
1.25d
1.7d
3.4d
12d
19d
35d

Supplementary Figure 7. Principal Component (**PC**) analysis on periodograms (N=37). (**a**) The six first **PC**s explain 98.7% of the variance of the periodograms, with a sudden fall of additional explained variance to less than 1% thereafter (red dot). Each of the first six **PC**s reveals a key aspect of periodicity in the entire data, and **PC**s are ranked by decreasing amount of explained variance. (**b**) **PC**#1 shows a very high peak at 28 days that accounts for 84.3% of the total variance, illustrating that monthly oscillations are prevalent in the data (note different y-axis ranges). (**c**) **PC**#2 accounts mostly for circadian and ultradian oscillations that were present in all subjects. (**d**) **PC**#3 accounts for the 3-week periodicity, and (**e**) **PC**#4 for the weekly and bi-weekly periodicities that frequently co-exist in the data. (**f**) Apart from recapitulating circadian and ultradian periodicities, **PC**#5 accounts for oscillations longer than one month. (**g**) **PC**#6 probably corrects 2-week or 3-week frequencies that are not exactly 14 or 21 days long.

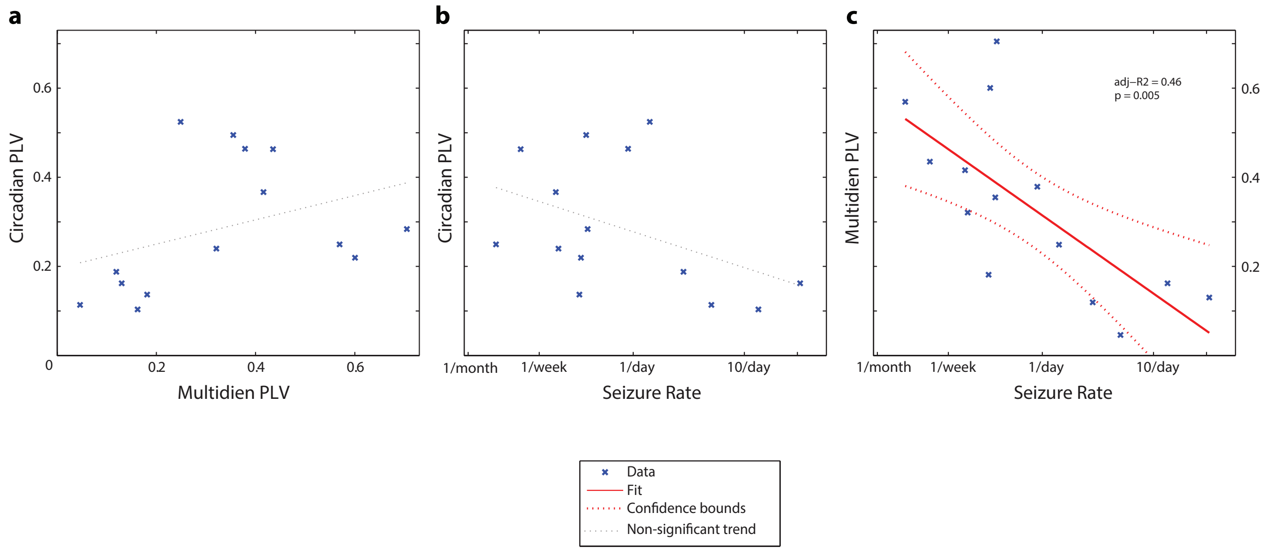

Supplementary Figure 8. Linear correlations between Phase-Locking Value (*PLV*) and seizure rate (N=14). (**a**) Non-significant correlation between circadian and multidien *PLV* (p = 0.2). (**b**) Non-significant correlation between circadian *PLV* and seizure rate (p = 0.1). (**c**) Significant correlation between multidien *PLV* and seizure rate (p = 0.005).

ID	Age	Sex	Catamenial	Etiology	Laterality	Localization	RNS Reason	Lead 1	Lead 2	Length [m]	Discard	Gaps	Valid Data [m]	Sz PPV
S1	39	M	-	Encephalitis	R	Mesiotemporal	Prior resection	R hpc	R laterotemp	27	1.5%	0	26.5	100%
S2	30	M	-	Idiopathic	B	Mesiotemporal	Bilaterality	R hpc	L hpc	22	7.7%	0	20.1	< 90%
S3	33	M	-	Idiopathic	B	Mesiotemporal	Bilaterality	L hpc	R hpc	25	2.1%	0	24.4	98%
S4	44	M	-	Idiopathic	B	Mesiotemporal	Bilaterality	L hpc	R hpc	23	2.3%	0	22.3	92%
S5	36	M	-	Infection	B	Mesiotemporal	Bilaterality	R hpc	L hpc	93	1.2%	34.3%	60.0	100%
S6	45	F	No	Birth trauma	B	Mesiotemporal	Bilaterality	R hpc	R laterotemp	103	0.5%	18.5%	83.6	< 90%
S7	43	M	-	Head trauma	B	Mesiotemporal	Bilaterality	R hpc	L hpc	94	1.0%	1.2%	92.1	100%
S8	45	M	-	Idiopathic	L	Laterotemporal	Language	L laterotemp	L laterotemp	22	9.9%	0	19.4	N<20
S9	25	M	-	Infection	B	Mesiotemporal	Bilaterality	R frontal	R frontal	119	1.2%	2.4%	114.6	100%
S10	58	M	-	Idiopathic	L	Laterotemporal	Language	L hpc	L laterotemp	100	0.5%	3.1%	96.8	< 90%
S11	40	F	No	Dysplasia	L	Frontal	Language	L frontal	L frontal	82	2.2%	20.9%	63.0	< 90%
S12	26	F	No	Infection	B	Mesiotemporal	Bilaterality	R hpc	L hpc	94	1.0%	3.4%	90.2	< 90%
S13	37	F	No	Idiopathic	R	Mesiotemporal	Prior resection	R hpc	R laterotemp	116	4.8%	22.7%	83.9	100%
S14	27	M	-	Vascular	L	Fronto-temporal	Prior resection	L frontal	L hpc	95	0.5%	2.2%	92.4	< 90%
S15	24	M	-	Dysplasia	R	Frontal	Motor	R frontal	R frontal	90	0.6%	0.1%	89.1	< 90%
S16	22	M	-	Idiopathic	L	Fronto-parietal	Motor	L parietal	L frontal	102	0.5%	4.7%	96.3	< 90%
S17	43	M	-	Head trauma	B	Mesiotemporal	Bilaterality	R hpc	L hpc	100	1.9%	25.4%	72.3	< 90%
S18	43	F	No	Vascular	L	Frontal	Motor	L frontal	L frontal	94	1.5%	12.4%	80.8	< 90%
S19	26	F	No	Infection	B	Mesiotemporal	Bilaterality	R laterotemp	R hpc	86	1.0%	27.9%	61.4	< 90%
S20	24	F	No	Dysplasia	L	Frontal	Motor	L frontal	L frontal	25	3.4%	18.5%	19.6	< 90%
S21	36	F	No	MTS	B	Mesiotemporal	Bilaterality	R hpc	L hpc	23	4.2%	0	22.0	100%
S22	38	M	-	Idiopathic	L	Frontal	Motor	L IH	L frontal	22	5.3%	0	21.1	< 90%
S23	41	M	-	Infection	B	Mesiotemporal	Bilaterality	R hpc	L hpc	118	6.2%	2.4%	107.9	< 90%
S24	40	F	No	MTS	B	Mesiotemporal	Bilaterality	R hpc	L hpc	41	0.5%	1.0%	40.4	93%
S25	27	F	No	Idiopathic	L	Fronto-parietal	Motor	L frontal	L parietal	95	0.5%	0.5%	93.8	< 90%
S26	21	M	-	Infection	B	Mesiotemporal	Bilaterality	R hpc	L hpc	95	0.0%	0.7%	94.3	100%
S27	28	M	-	Dysplasia	L	Laterotemporal	Language	L laterotemp	L laterotemp	18	8.9%	0	16.1	< 90%
S28	48	M	-	Idiopathic	B	Mesiotemporal	Bilaterality	L hpc	R hpc	14	3.7%	0	13.5	< 90%
S29	29	F	Yes	Dysplasia	R	Occipital	Vision	R occipital	R occipital	13	3.7%	0	12.3	100%
S30	54	F	No	MTS	B	Mesiotemporal	Bilaterality	R hpc	L hpc	12	10.3%	0	10.5	93%
S31	39	M	-	Idiopathic	L	Laterotemporal	Prior resection	L hpc	L laterotemp	11	11.0%	0	9.9	N<20
S32	22	F	No	Idiopathic	B	Insula	Bilaterality	L insula	R insula	9	6.4%	0	8.0	< 90%
S33	34	M	-	Head trauma	B	Mesiotemporal	Bilaterality	L hpc	R laterotemp	7	10.7%	0	6.6	100%
S34	33	F	No	Head trauma	B	Frontal	Bilaterality	L frontal	R frontal	6	22.1%	0	5.0	< 90%
S35	35	F	No	Idiopathic	L	Parietal	Language	L parietal	L parietal	6	1.4%	0	6.1	N<20
S36	20	M	-	Developmental	L	Laterotemporal	Language	L hpc	L parietal	6	47.0%	0.5%	3.1	< 90%
S37	43	M	-	Head trauma	B	Mesiotemporal	Bilaterality	L hpc	R hpc	5	10.0%	1.2%	4.7	100%

Supplementary Table 1. Subject characteristics and recording length. Subject ages are at time of RNS System implantation. Seizure lateralization, R: right, L: left, B: bilateral. Location of RNS System leads 1 and 2, hpc: hippocampus, laterotemp: lateral temporal neocortex. Length of recording and valid data analyzed in the present study are in months [m]. Discarded data and gaps in recordings expressed as percentage of total recording length. Positive Predictive Value (PPV) of LEA) as a surrogate for seizures. Individuals with LEA <20 or with PPV < 90% were not included for seizure phase analysis (gray, see Methods).
